# Container metaphor and the cognitive conventionalization of residential naming: a diachronic corpus study of the [X + Li] construction in Chinese

**DOI:** 10.3389/fpsyg.2026.1794229

**Published:** 2026-07-21

**Authors:** Danyang Zheng

**Affiliations:** School of Foreign Languages, Tianjin University, Tianjin, China

**Keywords:** constructional semantics, container metaphor, diachronic corpus analysis, linguistic landscape, metaphor-type distribution, residential naming

## Abstract

This study investigates the diachronic metaphorical organization of the Chinese residential naming construction [X + Li] from 1970 to 2019 from a linguistic landscape and cognitive-semantic perspective. Using a corpus of officially registered residential names from Tianjin collected from Anjuke, a major Chinese online real-estate listing platform, the study analyzes 1,672 [X + Li] instances and 228 [X + Yuan] instances. Metaphor types in the modifier X were annotated and inter-annotator reliability was assessed on stratified subsets of both corpora. The annotated data identify eight metaphor types grouped into four cognitive domains. Frequency profiling shows that state metaphors constitute the largest type within [X + Li], while residual analysis shows that the strongest contrast between [X + Li] and [X + Yuan] lies in the marked overrepresentation of category metaphors in [X + Yuan]. To visualize diachronic similarity among metaphor types, multidimensional scaling (MDS) is applied to decade-level proportional profiles. The MDS configuration shows that state metaphors have a distinctive diachronic proportional pattern, remaining highly recurrent across the five decade-based periods rather than reflecting a period-specific fluctuation. Overall, the findings support an interpretation of [X + Li] as a conventionalized spatial-metaphorical naming pattern grounded in a container schema, through which abstract states are regularly associated with bounded residential space.

## 1 Introduction

Linguistic landscape research has become an important perspective to understand the presentation of language in urban space and its social and cultural significance. Recent studies suggest that linguistic identification extends beyond mere language use to index underlying social structures, power relations, and forms of cultural consciousness embedded in urban space (Zhang and Zhang, 2024; Yang and Zhou, 2025). As a form of linguistic landscape, residential place names not only assume the basic naming function, but also classify and distinguish space through its stable and recurring naming mode, and construct a symbolic social space (Karpava, 2024; Blumenfeld and Cieślicka, 2025).

In the context of Chinese cities, the rapid urbanization process, the reform of the housing system and the reorganization of the socio-spatial structure have made significant changes in the practice of residential naming in the past few decades. These changes provide a crucial research opportunity to examine how spatial meaning is conventionalized in relatively stable naming constructions and continuously expanded in the time dimension.

In contemporary Chinese residential names, [X + Li] is a particularly prominent naming structure. “Li” has a long history of use in Chinese language. Its early meaning is not a place name in the general sense, but a grass-roots residential unit with clear boundaries and internal organization. In pre-Qin and Han literature, “Li” often appears as a settlement and management unit, referring to the neighborhood space formed by a certain number of households, and is closely related to household registration management, social organization and spatial division (Chen, 2020; Li, 2004; Wang, 2021)^1^. This usage makes “li” associated with “internal members,” “spatial boundaries” and “wholeness” from the beginning, rather than just representing abstract direction or position.

With the dissolution of the Li-fang (里坊) administrative system after the late Tang and early Song dynasties (Zhan, 1994), the word “Li” gradually lost its function as an institutional unit. However, its core spatial semantics—referring to an internally bounded residential domain—was retained and subsequently conventionalized in a more abstract form.

In modern Chinese, “Li” still retains the nominal function of the unit of living space, and on the other hand, it echoes the overlap of the locative morpheme 裡 (Li; in) representing “internal” in phonetics and writing, so that it is easier to activate the spatial concept of “internal-external” opposition (Lü, 1980; Wang, 1999; Fang, 2018). Against this background, [X + Li] has developed into a residential naming structure with strong productivity, which is composed of variable points X and “Li,” forming names such as “和谐里 (Hexie Li; Harmony Li)” and “安康里 (Ankang Li; Safe and healthy Li)”.

Such names are used to identify specific residential locations, but they may also involve a systematic pattern of semantic extension. Because “li” historically denotes a bounded residential unit and is phonologically and graphically associated with the locative morpheme “li” (inside), the [X + Li] construction may provide a spatial frame through which non-spatial concepts, such as states, values, and ideals, are interpreted in relation to a bounded residential domain.

The traditional urban living form represented by “里弄 (lilong)” residence provides a material basis for this conceptualization. Lilong architecture refers to a form of high-density urban housing that developed in modern Chinese cities. Characterized by narrow alleyways and rows of adjoining residential units, it embodies a living pattern that integrates traditional community organization with modern urban planning. The physical layout of lilong neighborhoods typically highlights enclosure, internal access, and spatial segmentation.

The inner-style communities usually adopt an enclosed layout, emphasizing the privacy of internal space, neighborhood relations and collective identity (Li, 2014; Zhang, 2018; Liang, 2018; Wang, 2021). This form of residence with clear boundaries and access control is also regarded as a typical “closed community” model in Chinese cities (Pow, 2009; Huang, 2006; Webster et al., 2006), and its physical boundaries are specified at the spatial level. The distinction between the inside and outside emphasized by the “container” image diagram.

Under the framework of cognitive semantics, containers are regarded as one of the most basic image schemas, which is not only used to organize the understanding of physical space, but also widely participates in the conceptualization of abstract fields such as state, social category and institutional membership (Lakoff and Johnson, 1980; Johnson, 1987; Hampe, 2005). In Chinese, the suffix of the place name “li” coincides with the locative word representing “inside” in phonetics and writing, making it easier for the [X + Li] structure to activate the container diagram, thus supporting the metaphorical extension beyond the specific spatial representation. Examples such as “Taikoo Li” in contemporary residential and commercial naming reflect the ability of this structure to integrate abstract concepts such as memory, identity and cultural identity while continuing the connotation of spatial boundaries.^2^

This article focuses on the semantic organization at the level of the naming structure, and its analysis is based on the distribution pattern presented by the residential naming practice in long-term use. By examining the systematic preference and constraints of different metaphor types in the [X + Li] construction, this article tries to reveal the conceptual constraints contained in the construction itself.

In order to distinguish constructional effects from the lexical attributes of the modifier X, this study introduces [X + Yuan] as a contrast construction. The contrast is not intended to distinguish metaphorical from non-metaphorical residential names. Both [X + Li] and [X + Yuan] can host metaphorical modifiers, including abstract evaluative terms denoting happiness, harmony, prosperity, or other desirable residential qualities. The purpose of the comparison is instead to examine whether the two suffix constructions show different preferences in the distribution of metaphor types.

Etymologically, “yuan” primarily denotes gardens, enclosed grounds, or landscape-oriented places (*Shuowen Jiezi*; cf. Qin legal texts from Shuihudi). Its historical usage is closely associated with garden-like, aesthetic, recreational, and culturally marked spaces. By contrast, “li” is historically associated with bounded residential units, internal organization, and the inside–outside opposition. Thus, both suffixes may participate in metaphorical residential naming, but they provide different constructional frames for organizing the semantic contribution of X.

The comparison between [X + Li] and [X + Yuan] therefore concerns metaphor-type preference rather than the presence or absence of metaphor. In other words, the purpose is not to argue that one construction is metaphorical and the other is not, but to examine whether the two constructions organize metaphorical modifiers differently, for example through the relative prominence of state metaphors in [X + Li] and category metaphors in [X + Yuan]. If the two constructions display systematic differences in metaphor-type distribution, such differences can be interpreted as evidence that suffix constructions contribute to the organization of residential naming meanings, rather than merely reflecting the lexical semantics of the modifier X.

Based on this design, the study addresses the following research questions:

*RQ1:* How are metaphor types distributed in the [X + Li] construction from 1970 to 2019?

*RQ2:* Which metaphor types show long-term stability or distinctive diachronic proportional profiles within [X + Li]?

*RQ3:* How does the metaphor-type distribution of [X + Li] differ from that of [X + Yuan], and what do these differences reveal about constructional preferences in residential naming?

Methodologically, this study annotates and classifies the metaphor types encoded in the modifier X on the basis of a diachronic corpus of Tianjin residential names, and combines this annotation with quantitative distributional analysis. MDS is used descriptively to visualize similarities among metaphor types based on their diachronic proportional profiles.

By modeling metaphorical organization at the level of naming structure, this paper provides new empirical evidence for the study of spatial metaphor, structural semantics and conceptual organization in linguistic landscape, and shows how to explore the generalization in naming practice using the diachronic corpus analysis method without exceeding the scope of explanation allowed by corpus evidence.

The present study adopts a cognitive-semantic perspective, but it does not claim to provide direct psycholinguistic evidence of individual mental representation. Rather, it treats recurrent distributional patterns in institutionalized residential naming as corpus-based evidence for conventionalized patterns of spatial conceptualization. The cognitive claims made in this paper are therefore interpretive and constructional in scope, not experimental or individual-processing claims. Although this study does not provide experimental psycholinguistic evidence, it contributes to psychology-oriented research on language and place by showing how recurrent public naming practices in the built environment encode and conventionalize place-related meanings, values, and expectations of residential well-being.

## 2 Relevant research and theoretical background

### 2.1 Spatial metaphor and container metaphor

Spatial metaphor is one of the most systematic and well-documented metaphor types in cognitive linguistics. A large number of studies show that human understanding of abstract concepts is often based on structured mapping of spatial experience, among which “container” is generally regarded as one of the most basic and generative image schemas (Lakoff and Johnson, 1980; Lakoff, 1987). Container metaphors usually contain structural elements such as “internal-external,” “boundary” and “inclusive,” which are not only used to conceptualize specific physical spaces, but also widely participate in the expression of abstract fields such as state, emotion, social category and institutional structure (Kövecses, 2000; Hampe, 2005).

Cross-language research further shows that container metaphors show highly stable conceptual functions in different languages, and their extension paths often gradually extend from physical space to psychological states, social identity and institutional membership (Kövecses, 2015). In Chinese, spatial metaphors are particularly highly productive. Expressions such as “在困境中 (in trouble),” “走出低谷 (out of the trough)” reflect the universal cognitive mechanism of understanding non-spatial concepts through spatial structure (Wang, 1999; Lü, 2005).

**Figure 1:**
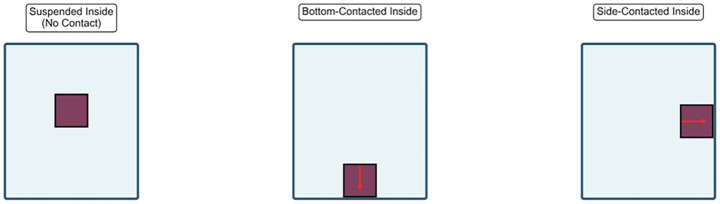
Spatial containment relationships for “Li” (Ge, 2004).

The spatial meaning of the contemporary Chinese character “里 (Li)” takes “the internal area surrounded by the boundary” as the core, and its container metaphor is mainly reflected in three basic constructions (see Figure 1), which jointly highlights the priority of internality relative to contact relationships.

It should be noted that container metaphor does not treat all spatial words equally. Different spatial terms have significant differences in the degree of prominence of “internality” and “boundaryness” in conceptual structure, which directly affects the direction and scope of their metaphorical expansion (Tyler and Evans, 2003; Hampe, 2005). Therefore, determining whether a naming construction has a stable spatial-metaphorical foundation requires an examination of its underlying conceptual structure, rather than relying solely on its surface-level spatial reference or nominal properties.

### 2.2 Constructional semantics, metaphorical compatibility, and distributional constraints

Constructive grammar emphasizes that the meaning in language is not entirely composed of words item by item, but there are structural units with overall semantics and functions (Goldberg, 1995; Croft, 2001; Goldberg, 2006). Constructions not only provide formal frameworks, but may also impose systematic constraints on the types of semantic content that can enter them, thereby shaping the range and acceptability of metaphorical extension (Boas, 2021; Dancygier and Sweetser, 2014).

Recent construction-based research suggests that the distribution of metaphorical uses is not arbitrarily generated, but is systematically licensed and constrained by the core meaning encoded in constructions, the semantics of constructional slots, and construction–frame alignment mechanisms. Constructions with more clearly defined semantic boundaries tend to exhibit stronger selectivity over the conceptual domains that may enter them, whereas constructions with more schematic semantics display greater “accommodative capacity,” allowing a wider range of metaphorical extensions (Perek and Hilpert, 2014). Accordingly, the frequency and stability of metaphor types within a given construction can be understood as distributional traces of the long-term operation of constructional semantics, rather than as mere rhetorical preferences.

From this perspective, examining the diachronic distribution of metaphor types carries dual theoretical significance. On the one hand, it reveals broad trends in metaphorical evolution over time; on the other hand, it allows us to infer, in reverse, the selection mechanisms by which constructions constrain metaphorical mapping at the conceptual level. If a particular metaphor type persistently and stably occurs within a specific construction across long-term usage, while other types show lower frequencies or more period-specific distributions, such structural asymmetries are more plausibly attributed to the semantic constraints of the construction itself than to incidental lexical preferences (Goldberg, 2006; Hilpert, 2021).

Conceptual integration theory provides a framework for explaining how elements from different input spaces can be selectively projected into a blended structure (Fauconnier and Turner, 2002). In the present study, it is used in a limited sense to account for the structural compatibility between the bounded residential-space schema of “Li” and abstract state concepts. This framework is not used to model online meaning construction, but to clarify why certain metaphorical combinations may be more compatible with the constructional semantics of [X + Li] than others.

### 2.3 Residence naming, linguistic landscape and conceptual organization

In place name research and linguistic landscape research, residence naming has long been regarded as a language practice with both referential function and social symbolism (Helleland, 2012; Karpava, 2024). Relevant research points out that residential names are not only used to identify specific spaces, but also to classify, separate and give connotation meaning to urban space through their recurring forms, so as to participate in the construction of social identity and spatial order (Relph, 1976; Tuan, 1977; Lefebvre, 1991).

The research on sociolinguistics and social place names has fully described the naming of Chinese residences from the perspective of naming motives, cultural symbols and ideologies (Tan, 2004). However, such research is mostly based on typology or descriptive classification, paying less attention to the distribution structure of different semantic types in specific naming structures, and lacking quantitative analysis based on large-scale time-laging corpus. As a result, some key questions have not been fully answered, such as whether different naming structures have systematic bias in the selection of metaphorical types? Does this bias remain stable in the time dimension?

The study of cognitive orientation provides a new perspective for understanding this problem. Relevant studies show that high-frequency, publicly visible language signs have the function of “environmental clues,” which can reflect the conceptual organization of space and identity of members of society without relying on individual subjective reports (Landry and Bourhis, 1997). In this sense, residential naming can be regarded as a long-term and stable language practice, and its distributional patterns can provide corpus-based evidence for conventionalized socio-cognitive and spatial-conceptual patterns.

### 2.4 The structural differences and theoretical significance of [X + Li] and [X + Yuan]

In the naming of Chinese residences, suffix forms such as “里 (Li),” “苑 (Yuan)”, “城 (Cheng)”, and “府 (Fu)” are highly active, but their differences in conceptual structure are often simplified to “place name suffixes” with similar functions. In fact, the focus of these suffixes on spatial conceptualization is significantly different.

“Li” refers to a residential unit with clear boundaries and internal organization at the etymology and structure level, and its semantic core is highly related to the “internal-external” opposition. This feature gives the [X + Li] construction a stable container-type spatial metaphorical basis, thus exerting potential constraints on the choice of metaphorical types.

The contrast between [X + Li] and [X + Yuan] is theoretically useful because it allows suffix meaning to be treated as a constructional factor rather than as a neutral place-name ending. Although both suffixes can occur in metaphorical residential names, they profile different aspects of spatial experience. Li foregrounds bounded residential interiority, whereas yuan foregrounds enclosed, garden-like, and aestheticized space. These different spatial profiles may make different kinds of metaphorical modifiers more compatible with each construction.

From this perspective, comparing [X + Li] with [X + Yuan] helps identify whether metaphor-type distribution is shaped by constructional semantics rather than by the lexical meaning of X alone. The comparison therefore serves as a control for evaluating how suffix-level spatial meaning contributes to the organization of residential naming patterns.

In summary, this study draws on spatial metaphor, constructional semantics, and linguistic landscape research to examine the conceptual organization of residential naming constructions in long-term public use. By analyzing the frequency, diachronic proportional patterns, and distributional similarity of metaphor types in the [X + Li] construction, and by comparing them with the [X + Yuan] construction, the study aims to identify constructional preferences in metaphor-type distribution. This theoretical stance provides the basis for the corpus-based methodology and the cautious interpretation of the empirical results.

## 3 Materials and methods

This study adopts a corpus-based diachronic analysis method to examine the distribution of metaphorical types and their changing characteristics in the long-term use of the residential naming structure [X + Li]. The core goal of the research is to reveal the systematic constraints on metaphorical selection at the structural level through large-scale natural corpus.

In terms of research and design, this study regards residential naming as an institutionalized, long-term stable language practice. Its metaphorical organization is not the improvised choice of individual names, but the result of gradual solidification in repeated use and social agreement. Therefore, the frequency, stability and distribution structure of different metaphor types in the time corpus can be regarded as effective clues to examine the semantic characteristics of conformations.

To distinguish construction-level semantic effects from the lexical properties of the modifier X, this study introduces [X + Yuan] as a contrast construction. By comparing differences in metaphor-type distributions between the two constructions, the analysis tests whether metaphor selection reflects systematic constructional biases rather than lexical variation.

### 3.1 Corpus source

The corpus used in this study was compiled from Anjuke,^3^ a large-scale real estate information platform in China. It includes residential names that were officially registered and put into use between 1970 and 2019, with a focus on constructions of the form [X + Li] and [X + Yuan]. In these constructions, X functions as a variable modifier, while “Li” and “Yuan” serve as the constructional cores.

As a comprehensive platform centered on residential transactions and property information, Anjuke primarily sources its residential naming data from official registration records and developer filings, ensuring a high degree of stability and standardization. The corpus includes only formal residential names with explicit referential functions and excludes commercial aliases, promotional labels, and unofficial abbreviations. Duplicate entries were systematically removed to prevent multiple counts of the same name.

To enable diachronic comparison, the corpus was further divided into five decade-based periods, allowing for an examination of changes in metaphor-type distributions over time as well as their relative stability across different historical stages.

### 3.2 Principles of division of metaphorical types

In order to examine the metaphorical organization in the [X + Li] structure, this paper classifies the metaphorical types embodied in the modified component “X.” The basic principle of classification is not to pursue detailed rhetorical distinction, but to pay attention to the degree of differences in spatial conceptualization of different types, that is, the compatibility between them and the spatial-container structure provided by “Li”.

In specific operations, according to the overall semantic characteristics of “X,” metaphorical types are divided into several categories, such as the type of state or nature, the type of social organization or system, and the type of psychological or activity process. The classification is based on the overall conceptual structure, not the superficial meaning of a single word.

To ensure transparency and replicability, a detailed annotation guide was prepared before coding and provided to the second annotator. The guide defined each metaphor type in terms of the semantic properties of X, diagnostic criteria, and representative examples. The final analytical model retained eight metaphor types, while the initial temporal category was excluded because only one temporal token occurred within the 1970–2019 [X + Li] dataset. The full coding scheme is provided in the Appendix.

To assess inter-annotator reliability, stratified subsets from both corpora were independently coded by a second annotator using the same classification criteria. For the [X + Li] corpus, 331 out of 1,672 tokens were selected; for the [X + Yuan] contrast corpus, 68 out of 228 tokens were selected, corresponding to approximately 30% of the dataset. The two annotation files were matched by residential name and completion year, and inter-rater agreement was calculated using both percentage agreement and Cohen’s kappa. As shown in Table 1, the agreement was high for both corpora: 89.12% with a Cohen’s kappa of 0.860 for [X + Li], and 88.24% with a Cohen’s kappa of 0.853 for [X + Yuan]. Disagreements between the two annotators were subsequently reviewed and resolved through discussion, and the agreed-upon labels were used in the final dataset. These results indicate a high level of annotation reliability.

**Table 1:** Inter-annotator reliability for the [X + Li] and [X + Yuan] annotation subsets.

Corpus	Total tokens	Reliability subset	Agreement	Cohen’s kappa
[X + Li]	1,672	331	89.12%	0.860
[X + Yuan]	228	68	88.24%	0.853

It should be emphasized that the present classification does not aim to provide an exhaustive or fine-grained typology of metaphorical meanings. Rather, it is an operational categorization motivated by the research question, namely, the degree to which different conceptual domains are compatible with the spatial container schema activated by “Li.” The categories are therefore defined at the level of overall conceptual structure, rather than lexical semantics of individual words, and are grounded in established distinctions in cognitive linguistics between spatial foundations, social organization, qualitative states, and internal dynamic processes (Lakoff and Johnson, 1980; Johnson, 1987; Kövecses, 2000; Hampe, 2005).

### 3.3 Diachronic distribution and statistical processing

After the completion of the metaphorical type annotation, this paper conducts a statistical analysis of the frequency of occurrence of different types in various periods of time to examine their stability and volatility in the time dimension. By comparing the distribution characteristics of different types in long-term use, it is possible to identify which metaphorical types are in the core position in the [X + Li] construction for a long time, and which types show marginal or staged characteristics.

At the same time, this study conducts a contingency-table analysis on the distribution of metaphorical types of [X + Li] and [X + Yuan] to test the systematic differences in metaphorical selection between the two constructions. Related statistical methods are used to describe the significance of distribution differences without involving the inference of the causal relationship between language use and psychology or behavior.

Different quantitative procedures were used for different analytical purposes. Raw frequency counts were used to describe the overall distribution of metaphor types and to conduct the chi-square comparison between [X + Li] and [X + Yuan]. For diachronic comparison, within-decade proportions were used because the number of tokens differed substantially across periods. For the MDS analysis, raw counts were converted into decade-level proportional profiles for each metaphor type, allowing the analysis to compare similarities among metaphor types in their proportional changes across the five decade-based periods rather than their absolute frequencies.

### 3.4 Multidimensional scaling analysis

To visualize similarity among metaphor types in their diachronic proportional patterns, this study applied multidimensional scaling (MDS) to decade-level distributional profiles. The analysis was conducted in Python using pandas, NumPy, SciPy, and matplotlib. The dataset was divided into five decade-based periods: 1970–1979, 1980–1989, 1990–1999, 2000–2009, and 2010–2019. For each decade, raw counts of metaphor types were converted into within-decade proportions, so that differences in corpus size across periods would not directly determine the comparison. Each metaphor type was therefore represented as a five-dimensional vector consisting of its proportional shares across the five decades.

Pairwise dissimilarities between metaphor types were calculated using Euclidean distance. Euclidean distance was selected because the analysis aimed to compare the overall similarity of metaphor types in their proportional changes across the same five decade-based dimensions. The distance between two metaphor types p and q was calculated as follows:

$$d(p,q)=\sqrt{\sum_{i=1}^{5}{\left(p_{i}-q_{i}\right)}^{2}}$$

Where p_i_ and q_i_ represent the within-decade proportions of metaphor types p and q in decade i. Smaller distances indicate more similar diachronic proportional profiles, whereas larger distances indicate more divergent proportional profiles.

The resulting 8 × 8 dissimilarity matrix was submitted to classical multidimensional scaling. Classical MDS was implemented through double-centering of the squared Euclidean distance matrix followed by eigenvalue decomposition, and a two-dimensional solution was retained for visualization. Model fit was evaluated using Stress-1, where lower values indicate better preservation of the original distance matrix in the reduced two-dimensional space.

The two MDS dimensions are latent mathematical coordinates generated to preserve pairwise dissimilarities. They are not predefined linguistic, semantic, or cognitive dimensions. The MDS configuration is therefore interpreted descriptively as a visualization of similarity among diachronic proportional profiles, rather than as a map of psychological distance or individual-level mental representation.

## 4 Results

### 4.1 Data overview and overall distribution of metaphor types

In the sorted corpus, a total of 1,672 [X + Li] instances and 228 [X + Yuan] instances were obtained, with a total of 1,900 residential names. The full dataset was first coded by the primary annotator, and stratified subsets from both corpora were independently coded by a second annotator for reliability assessment.

Eight metaphor types were identified and organized according to their main conceptual domains as shown in Table 2.

**Table 2:** Metaphorical types organized by cognitive domain.

Cognitive domain	Metaphor type	General semantic focus	*n* in [X + Li]	% in [X + Li]
Spatial foundation	Position	Specific spatial anchoring	127	7.6
	Scope	Non-specific spatial or natural range	320	19.1
Organizational-social	Institution	Institutional or administrative reference	30	1.8
	Category	Symbolic, cultural, or social category	85	5.1
Qualitative attributes	Atmosphere	Environmental or sensory ambience	228	13.6
	State	Abstract evaluative state or desirable condition	630	37.7
Internal-dynamic	Psychological perception	Affective or perceptual experience	9	0.5
	Activity	Action, process, or event	243	14.5
Total			1,672	100%

Table 2 summarizes the metaphor types according to cognitive domain, general semantic focus, and frequency distribution. Among them, the “Spatial Foundation” metaphor (Position, Scope) provides the most direct spatial reference resources; the “Organizational-social” category (Institution, Category) involves institutional and social classification; the “Qualitative Attributes” category (Atmosphere, State) mainly carries the concepts of integrity and state; the “Internal-dynamic” class (Psychological perception, Activity) reflects the internal experience or process characteristics.

**Figure 2:**
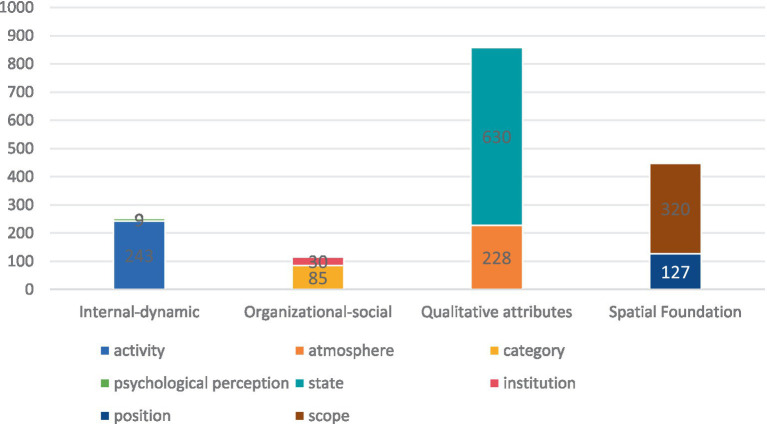
Frequency distribution of metaphor types across cognitive domains in the [X + Li] construction.

Figure 2 shows the overall distribution of metaphorical types in the [X + Li] corpus and is classified by four cognitive domains. The overall distribution shows significant structural asymmetry, and the proportional differences across cognitive domains are substantial.

Among them, “Qualitative Attributes” constitute the largest cognitive domain (*n* = 858), mainly driven by “state” metaphors (*n* = 630), and supplemented by “atmosphere” metaphors (*n* = 228). This distribution shows that [X + Li] naming practice obviously prioritizes coding the evaluation state of persistence—such as stability, happiness and collective value identity —over transient attributes or situational descriptions. The prominent position of this cognitive domain reflects a systematic tendency: that is, the conceptualization of living space as a container space that carries the quality of integrity and continuity.

“Spatial Foundation” constitutes the second largest category (*n* = 447), mainly composed of “scope” metaphor (*n* = 320) and “position” metaphor (*n* = 127). This distribution highlights the continuous importance of spatial anchoring, territorial reference and scale expansion in residential naming, indicating that the evaluative significance does not exist apart from the spatial structure, but is stably embedded in the identifiable spatial framework.

In contrast, the “internal-dynamic” domain is significantly smaller (*n* = 252), and it is almost entirely composed of “activity” metaphors (*n* = 243) and “psychological perception” metaphors only account for a very small proportion (*n* = 9). This structural imbalance shows that the status of time process and internal subjective experience in residential naming is relatively marginal, and naming practice is more inclined to stability and social sharing meaning than dynamic process or subjective experiential expression.

Finally, the “Organizational-social” domain is the smallest cognitive domain (*n* = 115), which is composed of “category” metaphor (*n* = 85) and “institutional” metaphor (*n* = 30). Although the social classification and institutional structure are reflected in the naming, their overall frequency is low, indicating that compared with the natural attributes and spatial basic dimensions, the [X + Li] naming is less dependent on the dominant social classification or organizational structure.

Overall, this distribution structure shows that the [X + Li] construction is strongly characterized by stable states and evaluative meanings at the system level and is supported by the spatial infrastructure, while the dynamic process meaning and the social-institutional meanings remain less frequent in the corpus. This distribution model provides quantitative evidence for the following explanation: [X + Li] construction mainly conceptualizes the living space as “container space that carries a lasting state and value,” rather than the place where the event occurs, the field of action or the institutional classification unit.

In the [X + Li] construction, the distribution of different metaphorical types is obviously uneven. Some metaphorical types account for a high proportion in the corpus, while others only appear sporadically. This imbalance is not concentrated in a certain period of time, but can be observed in the overall time span.

### 4.2 Comparison of the distribution of metaphorical types of [X + Li] and [X + Yuan]

To examine whether the two constructions show different metaphor-type distributions, this study conducted a contingency-table analysis of metaphor-type frequencies in [X + Li] and [X + Yuan]. The chi-square test indicates that the overall distribution of metaphor types differs significantly between the two constructions (χ^2^ = 47.696, df = 7, *p* < 0.001, Cramer’s V = 0.158), although the effect size is relatively modest.

As shown in Table 3, state metaphors constitute the largest category within [X + Li] (*n* = 630, 37.68%), followed by scope metaphors (*n* = 320, 19.14%) and activity metaphors (*n* = 243, 14.53%). In [X + Yuan], state metaphors also account for a substantial proportion (*n* = 67, 29.39%), but category metaphors are much more prominent than in [X + Li] (15.35% vs. 5.08%).

**Table 3:** Residual analysis for metaphor type comparison between [X + Li] and [X + Yuan].

Metaphor type	[X + Li] *n* (%)	Expected	Std. residual	Adj. residual	[X + Yuan] *n* (%)	Expected	Std. residual	Adj. residual
Activity	243 (14.53%)	242.9	0.0	0.02	33 (14.47%)	33.1	0.0	−0.02
Atmosphere	228 (13.64%)	224.4	0.2	0.75	27 (11.84%)	30.6	−0.7	−0.75
Category	85 (5.08%)	105.6	−2.0	−5.98	35 (15.35%)	14.4	5.4	5.98
Institution	30 (1.79%)	26.4	0.7	2.04	0 (0.00%)	3.6	−1.9	−2.04
Position	127 (7.60%)	121.4	0.5	1.51	11 (4.82%)	16.6	−1.4	−1.51
Psychological Perception	9 (0.54%)	7.9	0.4	1.11	0 (0.00%)	1.1	−1.0	−1.11
Scope	320 (19.14%)	330.0	−0.6	−1.77	55 (24.12%)	45.0	1.5	1.77
State	630 (37.68%)	613.4	0.7	2.44	67 (29.39%)	83.6	−1.8	−2.44
Total	1,672 (100%)	1,672.0			228 (100%)	228.0		

The adjusted residuals show where the main constructional differences lie. Category metaphors are significantly underrepresented in [X + Li] (adjusted residual = −5.98) and significantly overrepresented in [X + Yuan] (adjusted residual = +5.98), making this the strongest contrast between the two constructions. State metaphors show the opposite but weaker pattern: they are overrepresented in [X + Li] (adjusted residual = +2.44) and underrepresented in [X + Yuan] (adjusted residual = −2.44). Institution metaphors also show a weaker contrast, being relatively more associated with [X + Li] than [X + Yuan] (adjusted residuals = +2.04 and −2.04, respectively), although this result should be interpreted cautiously because the overall frequency of this type is low.

Overall, the comparison does not suggest that metaphorical naming is absent from [X + Yuan]. Rather, it shows that the two constructions organize metaphorical modifiers differently. [X + Li] is characterized by a high internal proportion of state metaphors and a significant relative association with this type, whereas [X + Yuan] shows a much stronger association with category metaphors. This contrast supports the argument that constructional semantics shapes metaphor-type preferences, rather than determining a simple metaphor/non-metaphor distinction.

### 4.3 Diachronic stability and distributional similarity of metaphor types

This section reports the MDS results based on the decade-level proportional profiles described in Section 3.4. The purpose of the analysis is to visualize similarities and differences among metaphor types in their diachronic proportional patterns. The model fit is first evaluated using Stress-1, followed by an interpretation of the heatmap and MDS configuration.

#### 4.3.1 Model fit of the MDS configuration

The goodness of fit of the MDS configuration was evaluated using Stress-1. In the revised analysis, each metaphor type was represented by a decade-level proportional profile, consisting of its within-decade proportions across the five periods: 1970–1979, 1980–1989, 1990–1999, 2000–2009, and 2010–2019. Pairwise dissimilarities between metaphor types were calculated using Euclidean distance. The resulting distance matrix was then submitted to classical multidimensional scaling, and a two-dimensional solution was retained for visualization.

Stress-1 measures the extent to which a two-dimensional MDS configuration preserves the original distance relationships in the input distance matrix. Lower values indicate better preservation of the original dissimilarity structure. As a general rule of thumb, Stress-1 values below 0.05 are often interpreted as indicating a very good fit, values around 0.10–0.20 indicate a moderate fit, and values above 0.20 suggest that the two-dimensional solution should be interpreted with caution.

The Stress-1 value of the present MDS solution is 0.029, indicating that the two-dimensional configuration preserves the original distance relationships among metaphor types very well. This suggests that the plotted configuration provides a reliable descriptive visualization of similarities and differences among metaphor types in their diachronic proportional patterns.

The two MDS dimensions are latent coordinates derived from the distance matrix and are used to represent pairwise dissimilarities among metaphor types. Accordingly, the interpretation focuses on relative distances among points rather than on assigning independent semantic labels to Dimension 1 or Dimension 2. In this study, the MDS plot is used as a descriptive visualization of similarity among diachronic proportional profiles.

#### 4.3.2 Time-based structural characteristics of metaphorical types

**Figure 3:**
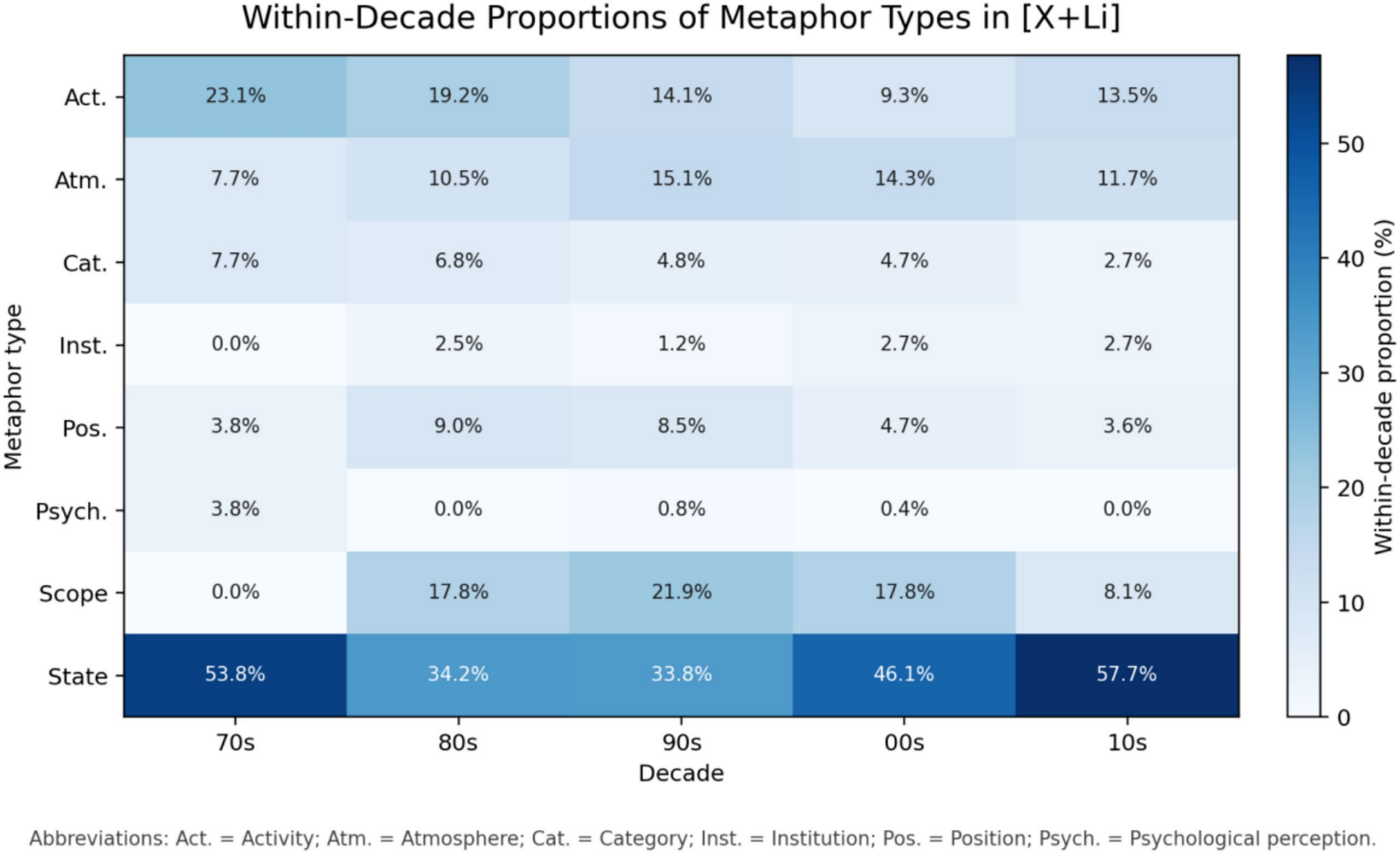
Diachronic distribution of metaphor types in the [X + Li] construction by decade.

Figure 3 presents the within-decade proportions of metaphor types in the [X + Li] corpus. This heatmap provides the direct empirical basis for interpreting diachronic change, because each cell shows the proportion of a given metaphor type within a specific decade. The results show that state metaphors remain the most prominent type across all five periods. They account for 53.8% of the 1970s subset, decline to 34.2% in the 1980s and 33.8% in the 1990s, and then rise again to 46.1% in the 2000s and 57.7% in the 2010s. This pattern indicates that state metaphors are not restricted to a single historical stage, but remain recurrent across the entire observation period.

Other metaphor types show more period-specific patterns. Activity metaphors decrease from 23.1% in the 1970s to 9.3% in the 2000s, before rising slightly to 13.5% in the 2010s. Scope metaphors are absent in the 1970s but become prominent from the 1980s to the 2000s, reaching 21.9% in the 1990s and 17.8% in both the 1980s and 2000s, before declining to 8.1% in the 2010s. Atmosphere metaphors show a gradual increase from 7.7% in the 1970s to 15.1% in the 1990s and 14.3% in the 2000s, followed by a slight decline in the 2010s. By contrast, institution and psychological perception remain consistently low across the five periods. These results suggest that, although the relative weight of individual metaphor types changes over time, the [X + Li] construction continues to favor state-related and spatially grounded meanings.

**Figure 4:**
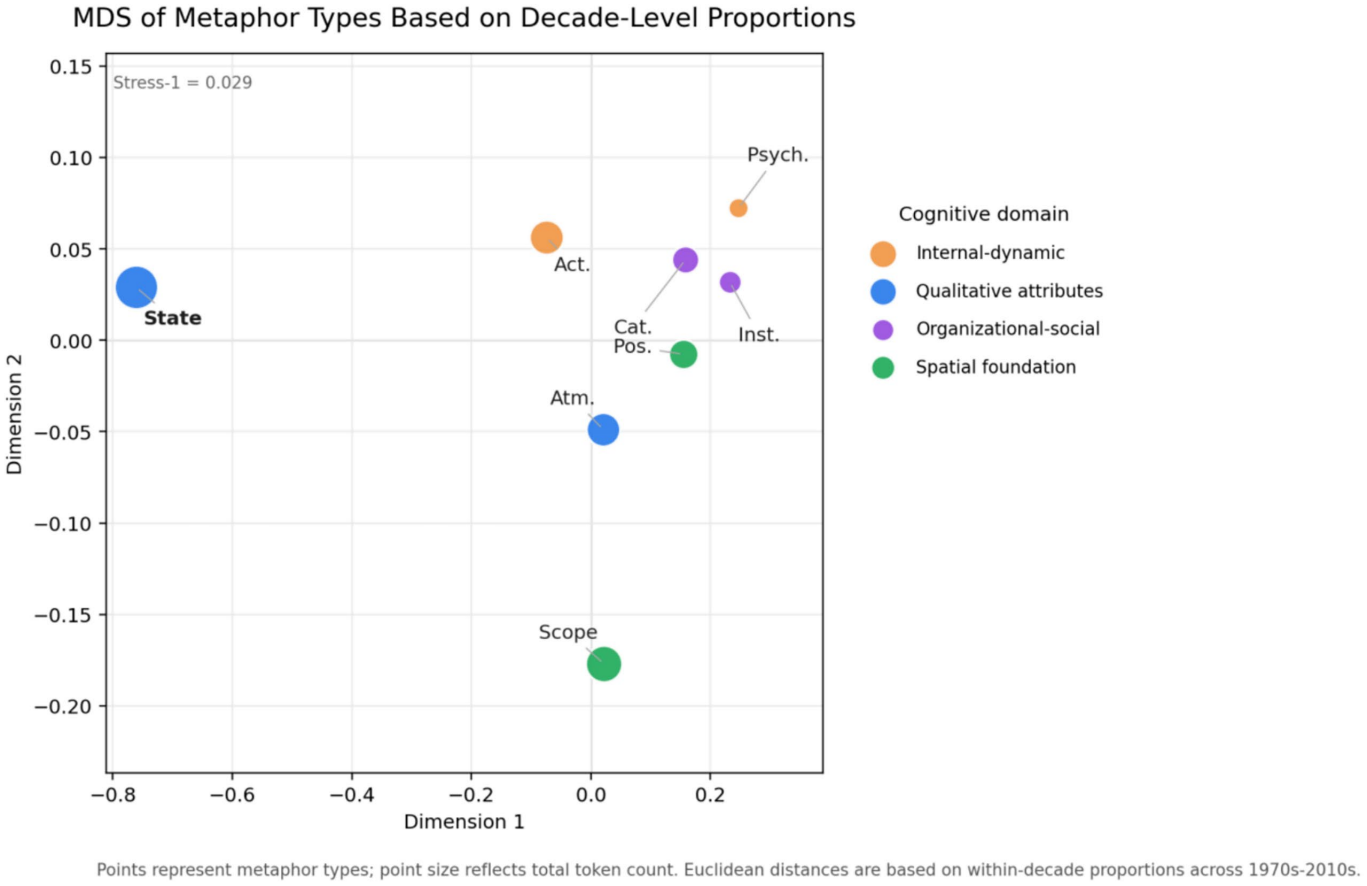
MDS configuration of metaphor types based on decade-level proportions.

Before interpreting the MDS results, it is necessary to clarify that Figure 4 is used descriptively. The distance between points does not represent psychological distance or cognitive centrality, but the degree of dissimilarity between metaphor types in their decade-level proportional profiles. Metaphor types located farther apart have more different diachronic proportional patterns, whereas those located closer together show more similar proportional changes across the five periods. Dimension 1 and Dimension 2 are therefore latent mathematical coordinates generated by the MDS procedure, rather than predefined semantic or cognitive dimensions.

Figure 4 complements the heatmap by summarizing the similarity among metaphor types based on their decade-level proportional profiles. Each point represents a metaphor type, and point size reflects the total token count of that type in the [X + Li] corpus. The MDS solution has a very low Stress-1 value of 0.029, indicating that the two-dimensional configuration preserves the original distance relationships well. It should be emphasized that Figure 4 does not display the proportion of each metaphor type in each decade directly; rather, it visualizes the overall similarity or dissimilarity among metaphor types on the basis of the proportional patterns shown in Figure 3.

The configuration shows that state metaphors are clearly separated from the other metaphor types. This separation does not mean that state metaphors are marginal in frequency or cognitive importance. Rather, it reflects their distinctive decade-level proportional profile. As shown in Figure 3, state metaphors remain the most frequent type across all five periods and are especially prominent in the 1970s, 2000s, and 2010s.

Scope and atmosphere are also positioned away from several other metaphor types in Figure 4, indicating that their overall proportional profiles across decades are relatively distinctive. The specific temporal patterns behind these positions are shown in Figure 3: scope metaphors are relatively prominent from the 1980s to the 2000s but decline in the 2010s, whereas atmosphere metaphors become more visible in the 1990s and 2000s. By contrast, category, institution, position, activity, and psychological perception are positioned closer to one another in Figure 4, suggesting more similar decade-level proportional profiles.

Taken together, Figures 3, 4 show two complementary aspects of diachronic structure. Figure 3 directly displays the within-decade proportion of each metaphor type, while Figure 4 summarizes the similarity among metaphor types based on these proportional profiles. The main finding is that state metaphors combine high frequency with a distinctive diachronic profile. This does not mean that MDS provides direct evidence of psychological distance or cognitive centrality. Rather, it shows that [state + Li] constitutes a stable and recurrent constructional pathway in the [X + Li] naming system, while other metaphor types show more limited, more fluctuating, or more period-specific distributions.

## 5 Discussion

The empirical findings of this study should first be understood as evidence of institutionalized naming regularities. Since the corpus consists of officially registered residential names, the patterns identified here primarily reflect conventionalized naming practices rather than direct observations of individual cognitive processing. Nevertheless, within a usage-based cognitive-semantic framework, recurrent and stable distributional patterns can be interpreted as corpus-based evidence for the conventionalization of spatial conceptualization at the level of public naming practice.

The following discussion interprets these corpus-based findings from three complementary perspectives. First, constructional semantics is used to explain how the [X + Li] construction constrains the metaphorical interpretation of its modifier X. Second, linguistic landscape research helps account for how residential names, as repeated and publicly visible signs, participate in the stabilization of socio-spatial meanings. Third, conceptual integration theory is used in a limited sense to explain the structural compatibility between the bounded residential-space schema of “li” and abstract state concepts. These perspectives are not treated as separate explanatory levels, but as complementary tools for interpreting the distributional patterns identified in the corpus.

### 5.1 Constructural differences, linguistic landscape and conceptual accessibility

The contingency analysis in Chapter 4 shows that there are significant differences in metaphor-type distribution between [X + Li] and [X + Yuan]. This difference is driven most strongly by the marked overrepresentation of category metaphors in [X + Yuan] and their underrepresentation in [X + Li]. At the same time, state metaphors constitute the largest category within [X + Li] and show a significant, though weaker, relative association with this construction. These findings suggest that metaphorical selection in residential naming is not a free combination of the modifier X, but is shaped by construction-specific semantic preferences.

From the perspective of linguistic landscape, this distribution difference can be understood as different constructions providing different environmental language inputs for conceptual understanding in public space. As a highly visible and recurring form of public language, residential names continue to enter the individual cognitive system without relying on explicit attention or subjective evaluation, thus affecting the accessibility and interpretation bias of concepts (Burwell and Lenters, 2015; Shohamy and Waksman, 2008). From a usage-based cognitive-semantic perspective, this long-term and institutionalized language input provides more stable activation conditions for certain conceptual structures, making it easier to be called when understanding living space.

In this sense, the structural differences between [X + Li] and [X + Yuan] are not only the opposition at the formal level, but also provide different degrees of cognitive usability for different types of concepts at the level of the linguistic landscape.

### 5.2 Usage-based cognition, structure regularization and metaphorical distribution stability

The diachronic stability of metaphor-type distributions in the [X + Li] construction—most notably the persistent high frequency of state metaphors across multiple decades—provides a prototypical illustration of usage-based models of cognition. Within the usage-based framework, linguistic structure is not conceived as a system of pre-stored abstract rules, but rather as a cognitive unit that gradually becomes entrenched through frequency effects in long-term use (Bybee, 2023; Ellis et al., 2015).

From this perspective, the sustained presence of “state metaphors” in the [X + Li] construction can be understood as the result of the entrenchment of form–meaning pairings through repeated usage. As Schmid (2020) argues, when a particular expression pattern recurs frequently within a stable contextual environment, its associated conceptual structure increasingly functions as a default interpretive pathway characterized by high predictability. The diachronic distributional stability identified in section 4 thus constitutes distribution-level evidence of this cognitive entrenchment process.

### 5.3 Socio-historical triggers of semantic conventionalization

The stability of state metaphors in the [X + Li] construction should be interpreted against the broader socio-historical transformation of Chinese urban housing. Previous studies have shown that China’s urban housing system underwent a profound shift from welfare-oriented allocation to market-oriented and commodified provision (Ping Wang and Murie, 1996; Huang and Clark, 2002; Chen and Wen, 2017). This transformation was accelerated by the 1994 State Council Decision on Deepening the Reform of the Urban Housing System and the 1998 policy terminating in-kind housing allocation, which marked a transition from state- and work-unit-based housing provision toward monetized housing distribution and market-oriented residential development. More recent studies further show that the post-reform housing market was closely linked to land finance, urban land marketization, and local-government-led urban expansion (Huang and Chan, 2018; Fan et al., 2020; Gyourko et al., 2022).

A methodological caution is necessary when interpreting the diachronic distribution of tokens in this study. The decade-based counts reported here do not represent the total number of residential compounds built in Tianjin in each decade. Rather, they reflect the distribution of currently retrievable second-hand residential community names on Anjuke that conform to the [X + Li] pattern. Therefore, the concentration of [X + Li] tokens in the 1990s should be understood as a corpus-specific pattern rather than as a direct measure of housing construction volume or naming productivity in that decade.

With this caution in mind, the corpus pattern can still be situated within the broader socio-historical transformation of Chinese urban housing. Housing reform altered the social meanings attached to residential space: housing increasingly became associated not only with allocation and residence, but also with property, community identity, life quality, security, prosperity, and symbolic value. Against this background, the stability of state metaphors across the five decade-based subsets becomes especially meaningful. It does not suggest that the social meaning of housing remained unchanged. Rather, it indicates that the [X + Li] construction provided a stable container-like semantic frame through which changing residential values could be expressed. In the welfare-housing context, state-related modifiers could be associated with stability, order, and settlement; in the market-oriented context, they could further encode harmony, prosperity, security, and desirable quality of life.

### 5.4 Distinctive diachronic profile of state metaphors

Multidimensional scaling reveals that state metaphors have a distinctive diachronic proportional profile, while their structural location remains highly stable. This distinctive diachronic profile should be interpreted within a usage-based cognitive-semantic framework.

Cognitive research suggests that the functional role of a concept is not determined solely by its structural centrality, but also by its recurrence, predictability, and contextual availability (Barsalou, 2008). In the present corpus, state metaphors constitute a recurrent and highly visible naming pattern in the [X + Li] construction, while maintaining a proportional profile that differs from those of other metaphor types. This pattern suggests that state-related meanings are not incidental additions to the construction, but form a stable pathway through which residential space is associated with abstract qualities, values, and desirable conditions.

From a usage-based perspective, this distributional pattern is compatible with the view that recurrent linguistic input can contribute to the conventionalization of particular form–meaning pairings over time (Schmid, 2020).

### 5.5 Cognitive mechanism of conceptual integration and selective projection

On the basis of the above-mentioned use of the basic cognitive and linguistic landscape framework, conceptual integration theory is introduced in this article as a supplementary analysis tool to explain why the naming model [state + Li] has long-term feasibility in cognitive structure and can become routinized through repeated use. It should be emphasized that this study does not regard the conceptual integration theory as a psychological modeling of the process of individual online meaning construction, but uses it to reveal the compatibility and projection selection mechanisms of different conceptual domains at the structural level.

**Figure 5:**
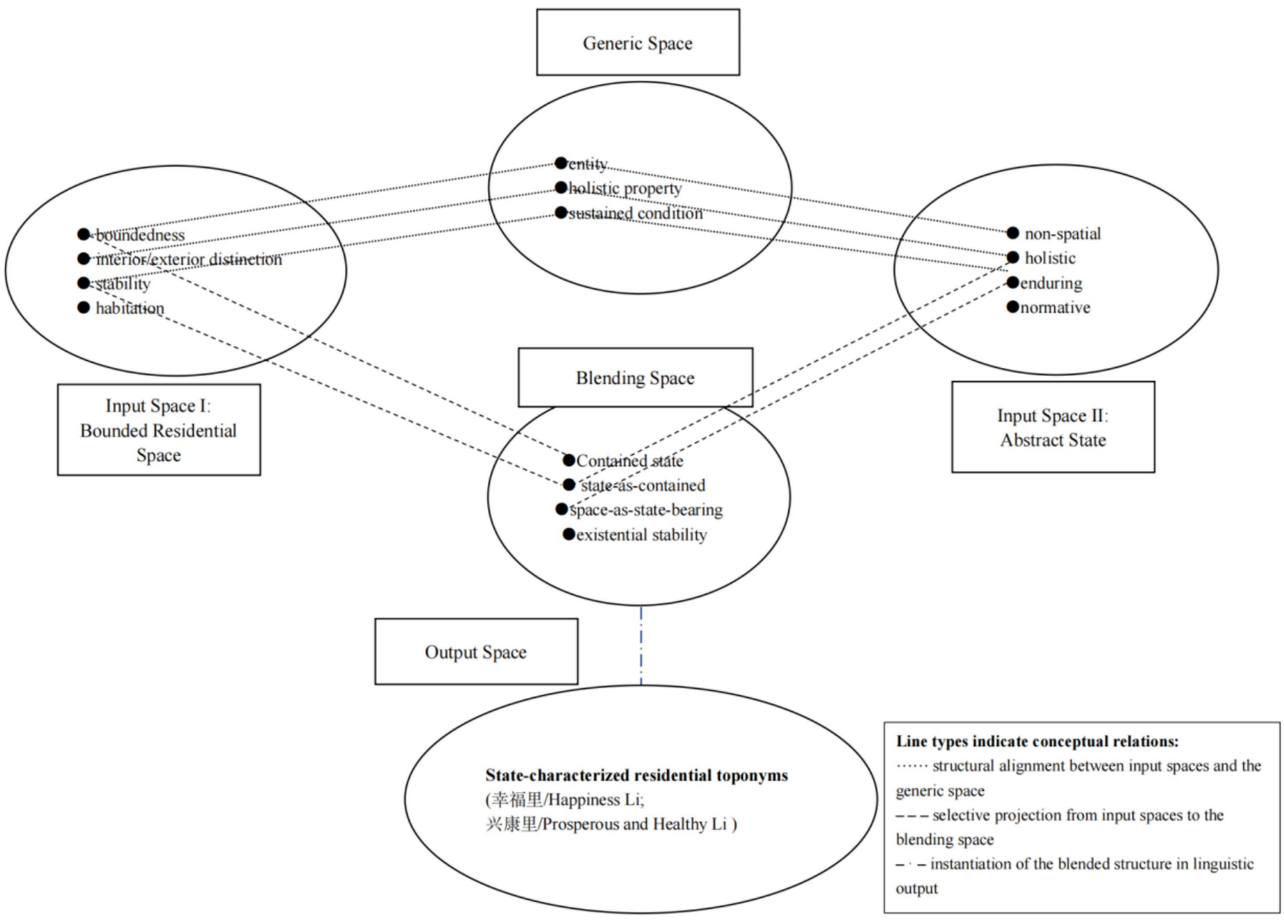
Conceptual blending construction underlying state-characterized [X + Li] residential toponyms.

As illustrated in Figure 5, the naming pattern [state + Li] can be analyzed as a process of conceptual blending between two input spaces. Input Space I corresponds to a bounded residential-space schema centered on “Li,” whose salient features include boundaryhood, an inside–outside distinction, stability, and habitability. Input Space II corresponds to abstract state concepts (e.g., happiness, well-being), whose semantic properties are characterized by non-spatiality, holistic structure, temporal continuity, and normative evaluation.

At a higher level of abstraction, the two input spaces share a common structural core—namely, entities conceived as wholes, holistic attributes, and sustained states. These shared structures are aligned in the generic space, enabling the structural compatibility that supports subsequent integration.

On this basis, not all attributes in the input space will enter the integrated space. Conceptual integration theory points out that projection is not a linear mapping, but a selection process subject to structural compatibility and cognitive constraints (Turner, 2020; Dancygier and Vandelanotte, 2025). As shown by the dotted line in Figure 5, only attributes that are highly fit at the schema level—most notably “holistic entityhood,” “temporal continuity,” and “states” that can be accommodated with a bounded domain--are projected into the blending space. By contrast, features that lack such structural compatibility, or that are redundant with respect to the emergent blend, are systematically backgrounded rather than transferred (Schmid, 2020).

In the blending space, a composite conceptual structure emerges in which a residential space is characterized by a particular state. Its core features include the accommodation of a state, the attribution of state-like properties to space, and existential stability at the level of being. This blended structure is subsequently instantiated in the output space, taking the form of concrete linguistic expressions such as 幸福里 (Xingfu Li; Happiness Li) and 兴康里 (Xingkang Li; Prosperous and Healthy Li), which exemplify state-characterized residential toponyms.

From a cognitive-semantic perspective, this projection pattern can be interpreted as the principle of cognitive economy in conceptual integration: attributes that are highly holistic and temporally stable are more readily integrated with existing spatial schemas than dynamic or processual features, and therefore require lower interpretive cost (Divjak et al., 2022). Consequently, the long-term predominance of “state metaphors” in the [X + Li] construction should not be attributed to the subjective preferences of individual name-givers, but rather to general cognitive constraints operating under conditions of sustained language use and repeated exposure within the linguistic landscape.

### 5.6 Spatial metaphor conventionalization in the linguistic landscape

When situated within a linguistic landscape perspective, the above analysis allows for a clearer understanding of the cognitive significance of the [state + Li] naming pattern. As a highly institutionalized form of public language, residential toponyms function as recurrent public linguistic input within the linguistic landscape, constituting a sustained form of environmental linguistic input (Cenoz and Gorter, 2022). Under such conditions, conceptual integration patterns that satisfy constraints of structural compatibility and cognitive economy are more likely to be reinforced and maintained through repeated use (Schmid, 2020; Karpava, 2024).

The distinctive position of state metaphors in the MDS configuration reflects their distinctive diachronic proportional profile and provides distributional support for this cognitive-semantic interpretation. This pattern indicates that [state + Li] is not an incidental variant within the construction, but rather a specialized projection pathway that has emerged and persisted through long-term naming practices. It is consistent with a process of cognitive conventionalization in which abstract states are systematically spatialized within a container-based conceptual structure.

Together, these findings directly address the three research questions outlined in the Introduction. Overall, this chapter demonstrates that metaphor distributions within the [X + Li] construction are not accidental outcomes, but the result of the long-term interaction between constructional semantics, usage frequency, and environmental linguistic input. As a highly stable naming pattern, [state+Li] has become a conventionalized spatial-metaphorical pattern within the linguistic landscape, functioning as a recurrent constructional resource for interpreting residential space. This finding provides corpus-based evidence for understanding the relationship between linguistic landscapes, constructional meaning, and spatial conceptualization from a usage-based cognitive-semantic perspective.

## 6 Conclusion

This study examined the diachronic metaphorical organization of the [X + Li] construction in Tianjin residential names. The findings answer the three research questions in the following ways.

First, the diachronic distribution shows that metaphor types in the [X + Li] construction did not remain static from 1970 to 2019. State metaphors remained the most prominent type across all five decade-based periods, while other types, such as scope, atmosphere, and activity, showed more period-specific fluctuations. This indicates that the construction combines long-term stability with historical variation in metaphor-type distribution.

Second, the MDS analysis shows that metaphor types differ in their diachronic proportional profiles. State metaphors are separated from other types in the MDS configuration, not because they are cognitively marginal, but because their proportional pattern across decades is distinctive. This supports the view that [state + Li] is a stable and recurrent constructional pathway within institutionalized residential naming practices.

Third, the comparison between [X + Li] and [X + Yuan] shows that the two constructions do not differ simply in whether they allow metaphorical naming. Rather, they differ in metaphor-type preferences. [X + Li] is characterized by the high internal proportion and diachronic recurrence of state metaphors, whereas the strongest constructional contrast lies in the marked overrepresentation of category metaphors in [X + Yuan]. This contrast suggests that residential naming suffixes can shape the distributional organization of metaphorical modifiers.

Several limitations should be acknowledged. The corpus consists of currently retrievable residential community names from Anjuke rather than a complete official registry of all Tianjin residential compounds. In addition, the analysis is corpus-based and distributional, and therefore does not provide direct psycholinguistic evidence of individual processing or mental representation. Future research could extend the comparison to other cities, other residential naming constructions, and additional socio-historical variables.

The psychological relevance of the study therefore lies not in modeling individual online language processing, but in examining how public linguistic forms in the built environment participate in the conventionalization of spatial meanings, residential values, and place-related conceptualization. Taken together, these findings contribute to research on spatial metaphor, constructional semantics, and linguistic landscape by showing how metaphorical meanings are conventionalized in public naming practices over time. Methodologically, the study demonstrates how corpus annotation, inter-annotator reliability testing, diachronic proportion analysis, and MDS visualization can be combined to examine constructional meaning without treating distributional patterns as direct evidence of individual psychological processing. While the present study does not provide psycholinguistic evidence of mental representation, it offers corpus-based evidence for conventionalized patterns of spatial conceptualization in institutionalized residential names.

## Data Availability

The raw data supporting the conclusions of this article will be made available by the authors, without undue reservation.

## Footnotes

1 The household-registration and local administrative arrangement referenced here is commonly termed the Li-fang system, a grid-like urban governance structure that organized residents into territorially bounded units for purposes of registration, policing, and everyday administration.

2
http://www.heb.chinanews.com.cn/zgxwzk/20250312452019.shtml

3 Anjuke: https://www.anjuke.com/

## Appendix

Table A1.

**Table A1:** Annotation criteria for metaphor-type classification.

Metaphor type	Definition	Diagnostic criteria	Examples
Position	X refers to a specific geographical location.	X is a proper place name, such as a city, province, river, historical place name, street, or other identifiable geographical entity.	延庆里 (*Yanqing Li*); 苏州里 (*Suzhou Li*); 宜昌里 (*Yichang Li*)
Scope	X refers to a non-specific natural or spatial range.	X denotes a general natural-geographical concept without a specific referent, such as mountain, water, lake, forest, or park-like spatial range.	环湖里 (*Huanhu Li; Ring-Lake Li*); 龙泉里 (*Longquan Li; Dragon-Spring Li*); 静园里 (*Jingyuan Li; Tranquil-Garden Li*)
Institution	X refers to an organization, institution, industry, or administrative function.	X contains institutional, occupational, administrative, or functional meaning.	银行里 (*Yinhang Li; Bank Li*); 航天北里 (*Hangtian Beili; Aerospace-Institute North Li*); 建材里 (*Jiancai Li; Building-Company Li*)
Category	X refers to a symbolic, cultural, or social category.	X denotes symbolic signs, cultural symbols, collective labels, or person-related categories.	龙凤里 (*Longfeng Li; Dragon-Phoenix Li*); 文化里 (*Wenhua Li; Culture Li*); 红旗里 (*Hongqi Li; Red-Flag Li*)
Atmosphere	X evokes an environmental, sensory, or atmospheric quality.	X refers to natural imagery, light, weather, seasonal, celestial, or plant-related elements that create an abstract environmental ambience.	曙光里 (*Shuguang Li: Drawn Li*); 星辰里 (*Xingchen Li; Star Li*); 晨光里 (*Chenguang Li; Morning-Light Li*)
State	X denotes an abstract state, quality, value, or desirable condition.	X is an adjective-like or noun-like abstract concept expressing auspiciousness, harmony, stability, happiness, virtue, or other evaluative states.	德安里 (*De’an Li; Virtuous-Peace Li*); 祥和里 (*Xianghe Li; Auspicious-Harmony Li*); 五福里 (*Wufu Li; Five-Blessings Li*)
Psychological perception	X denotes human perception, emotion, or affective experience.	X refers specifically to feeling, affection, perception, mental experience, or interpersonal sentiment.	怡心里 (*Yixin Li; Pleasant-Heart Li*); 友爱里 (*You’ai Li; Friendship Li*); 敬爱里 (*Jing’ai Li; Respectful-Love Li*)
Activity	X denotes an action, process, or event.	X is verbal or verb-like and expresses behavior, collective action, construction, celebration, or movement.	迎新里 (*Yingxin Li; Welcoming-New Li*); 自建里 (*Zijian Li;* Self-Built Li); 团结里 (*Tuanjie Li; Unity Li*)
